# Male-Specific Differences in Proliferation, Neurogenesis, and Sensitivity to Oxidative Stress in Neural Progenitor Cells Derived from a Rat Model of ALS

**DOI:** 10.1371/journal.pone.0048581

**Published:** 2012-11-08

**Authors:** Ruojia Li, Rachel Strykowski, Michael Meyer, Patrick Mulcrone, Dan Krakora, Masatoshi Suzuki

**Affiliations:** 1 Department of Comparative Biosciences, University of Wisconsin-Madison, Madison, Wisconsin, United States of America; 2 The Stem Cell and Regenerative Medicine Center, University of Wisconsin-Madison, Madison, Wisconsin, United States of America; University of South Florida, United States of America

## Abstract

Amyotrophic Lateral Sclerosis (ALS) is a fatal neurodegenerative disease characterized by progressive motor dysfunction and the loss of large motor neurons in the spinal cord and brain stem. A clear genetic link to point mutations in the superoxide dismutase 1 (SOD1) gene has been shown in a small group of familial ALS patients. The exact etiology of ALS is still uncertain, but males have consistently been shown to be at a higher risk for the disease than females. Here we present male-specific effects of the mutant SOD1 transgene on proliferation, neurogenesis, and sensitivity to oxidative stress in rat neural progenitor cells (rNPCs). E14 pups were bred using SOD1^G93A^ transgenic male rats and wild-type female rats. The spinal cord and cortex tissues were collected, genotyped by PCR using primers for the SOD1^G93A^ transgene or the male-specific *Sry* gene, and cultured as neurospheres. The number of dividing cells was higher in male rNPCs compared to female rNPCs. However, SOD1^G93A^ over-expression significantly reduced cell proliferation in male cells but not female cells. Similarly, male rNPCs produced more neurons compared to female rNPCs, but SOD1^G93A^ over-expression significantly reduced the number of neurons produced in male cells. Finally we asked whether sex and SOD1^G93A^ transgenes affected sensitivity to oxidative stress. There was no sex-based difference in cell viability after treatment with hydrogen peroxide or 3-morpholinosydnonimine, a free radical-generating agent. However, increased cytotoxicity by SOD1^G93A^ over-expression occurred, especially in male rNPCs. These results provide essential information on how the mutant SOD1 gene and sexual dimorphism are involved in ALS disease progression.

## Introduction

Amyotrophic lateral sclerosis (ALS) is a neurodegenerative disease characterized by the loss of the upper and lower motor neurons, resulting in progressive paralysis and eventually death. About 90% of ALS cases are sporadic and the remaining 10% of ALS cases are familial (FALS). Mutations in several genes have now been identified which cause ALS (reviewed in [1]). In approximately 20% of FALS cases, the cause can be attributed to a mutation in the Cu^2+/^Zn^2+^ superoxide dismutase 1 (SOD1) gene, a ubiquitously-expressed free-radical scavenger enzyme [2]. Over-expressing the human SOD1 mutant in rodents results in a disease progression similar to that observed in ALS patients, providing a valuable model (SOD1^G93A^ rodents) on which a great deal of ALS research has been based [3], [4]. Given the diversity of the physiological functions affected by genetic abnormalities, it is generally assumed that ALS is the result of defects in multiple cellular mechanisms [5].

The exact etiology of sporadic ALS is largely unknown, but epidemiological studies have shown that both the incidence and prevalence of ALS are greater in men than in women [6]. The male/female ratio of ALS occurrence is 4∶1 when age at onset is in the second decade, but this ratio declines steadily to 1∶1 at ages above 60 years [7]. The onset of the disease is also earlier for men than it is for women [8]. Furthermore, women who develop ALS have often had a later menarche and earlier menopause than healthy controls [9]. In agreement with these epidemiological studies of ALS patients, sex does affect the clinical course of the disease in transgenic rodent models for familial ALS using the mutant human SOD1 gene [10]–[14]. It has been suggested that sex steroids are involved in the occurrence or disease progression of ALS [10], [14]. However, we recently reported that the sexual dimorphism observed in the rat model of ALS may not be regulated by gonadal steroids [11]. Therefore, an interaction between sex and clinical phenotype in ALS is still a mystery. Understanding the causes of the sex differences in ALS could give clues to variables that modify the disease.

In this study, we used neural progenitor cells (NPCs) as a simple culture model to further explore the sex difference in ALS. Recent studies have shown that NPCs can be derived and cultured from the developing brain, allowing the direct observation of the proliferation, differentiation, and migration of these cells [15]–[17]. NPCs can be isolated from rodent and human fetal tissues and maintained in culture as spherical aggregates of undifferentiated cells termed neurospheres [18]–[21]. Here we prepared male and female rat NPCs (rNPCs) derived from fetal central nervous system (CNS) of SOD1^G93A^ transgenic rats. We then examined how sex and mutant SOD1 over-expression affected proliferation, neurogenesis and sensitivity to oxidative stress in culture.

## Materials and Methods

### Rat Neural Progenitor Cell Culture

Rat neural progenitor cells (rNPCs) were prepared from fetal brains and induced to proliferate as neurospheres using established passaging methods to achieve optimal cellular expansion as previously described in detail [19], [21]–[24]. The experimental protocol was approved by the University of Wisconsin-Madison Graduate School Animal Care and Use Committee (Protocol Number: G00515 and G00623). This study was carried out in strict accordance with the recommendations in the Guide for the Care and Use of Laboratory Animals of the National Institutes of Health.

**Figure 1:**
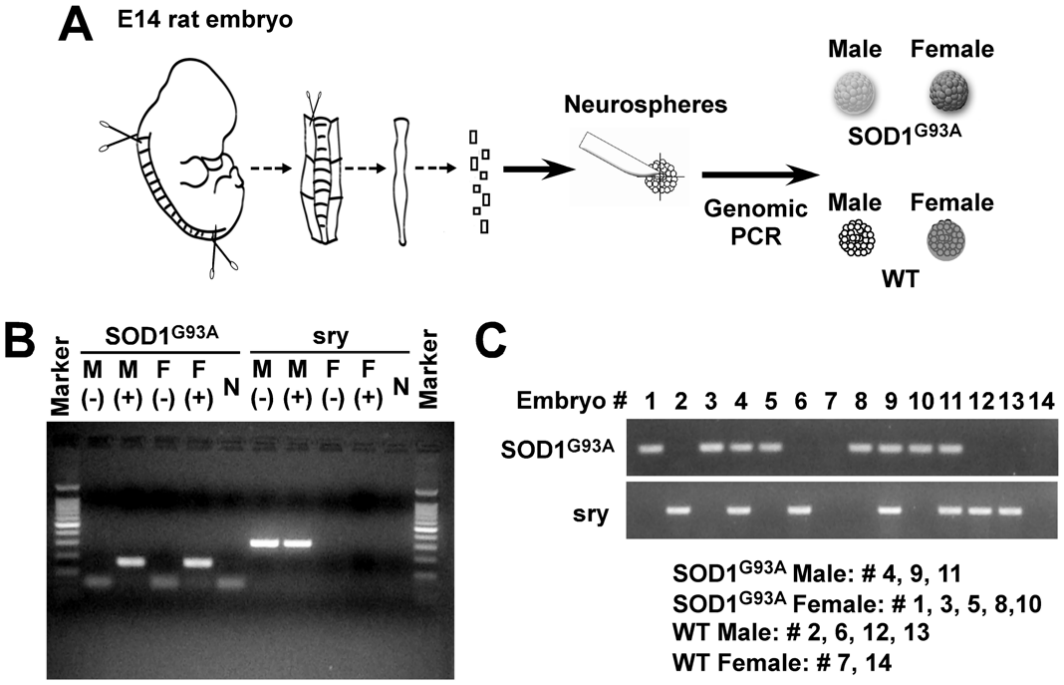
Generation of neural progenitor cells expressing mutant SOD1. (**A**) Schematic of the dissection method and preparation of four neurosphere lines: SOD1^G93A^ positive, negative (Wild type, WT), male, or female. (**B**) Genomic PCR using adult tail samples. (**C**) Selection of individual embryos by genomic PCR using the specific primers for SOD1^G93A^ or *Sry* gene.

Heterozygous SOD1^G93A^ males were mated with Sprague-Dawley wild type females to prepare pups for this study. According to our previous results [11], [12], we expected a stable 50% positive heterozygous offspring in each generation and approximately equal numbers of males and females. Rat fetuses at embryonic day 14 (E14) were harvested from pregnant rats, and their entire spinal cord and cortex were dissected. To euthanize these pregnant rats, sodium pentobarbital was used for terminal general anesthesia. All efforts were made to minimize suffering. The tissue from each pup was genotyped by genomic PCR using genomic DNA isolated from tail samples with primers for the SOD1^G93A^ transgene (forward primer, 5′-GTG GCA TCA GCC CTA ATC CA-3′; reverse primer 5′-CAC CAG TGT GCG GCC AAT GA-3′) or the male specific *Sry* gene (forward primer, 5′-TAC AGC CTG AGG ACA TAT TA-3′; reverse primer 5′-GCA CTT TAA CCC TTC GAT TGA-3′). All primers were obtained from Integrated DNA Technologies (Coralville, IA). We formed four groups: 1) male SOD1^G93A^ (−), 2) male SOD1^G93A^ (+), 3) female SOD1^G93A^ (−), and 4) female SOD1^G93A^ (+) (Fig. 1A). We prepared four pregnant females and used at least three independent rNPC lines in each group throughout this study. The collected tissues were then pooled and chopped by a McIlwain tissue chopper (Mickle Laboratory Engineering, Surrey, England) into 200 µm small cubes before being placed in maintenance medium [Dulbecco's modified Eagle medium (DMEM)/Hams F12 (7∶3) containing penicillin/streptomycin/amphotericin B (PSA, 1% v/v)] supplemented with B27 (2% v/v; Invitrogen, Carlsbad. CA), epidermal growth factor (EGF; 20 ng/ml; Sigma-Aldrich, St. Louis, MO), and fibroblast growth factor-2 (FGF-2; 20 ng/ml; R&D systems Inc., Minneapolis, MN) with heparin (5 µg/ml; Sigma-Aldrich). All cultures were maintained in a humidified incubator (37°C, 5% CO_2_ in air) and half of the growth medium was replenished every 3 to 4 days. Neurospheres were not allowed to grow any larger than 500 µm in diameter and were sectioned into 200 µm diameter spheres.

### Cumulative 5-Bromo-2′-demoxyuridine (BrdU) Pulse Labeling

The cumulative 5-Bromo-2′-demoxyuridine (BrdU)-labeling method has been used to identify newly dividing cells in neurospheres as previously described [19], [24]. Twenty to thirty neurospheres were labeled with 0.2 µM BrdU (Sigma-Aldrich) for 14 hours, and then dissociated into a single cell suspension with Accutase (Innovative Cell Technologies, San Diego, CA) for 10 minutes at 37°C. The resulting cells (25,000 cells/50 µl) were plated to glass coverslips coated with poly-L-lysine and laminin (Sigma-Aldrich) and cultured in the plating medium [DMEM/Hams F12 (7∶3) containing PSA with 2% B27 supplement (Invitrogen)] for 1–2 hour at 37°C before fixation. For differentiation studies, dissociated cells were continuously maintained in the plating medium and differentiated for 7 days, which allowed the dissociated cells to differentiate in serum-free conditions [19], [24].

### Immunocytochemistry

The cells were fixed in ice cold methanol for 10 min or 4% paraformaldehyde (PFA) for 20 min at room temperature, and washed in phosphate-buffered saline (PBS). For BrdU staining, the cells were then incubated in 2 M hydrochloric acid for 20 min at 37°C. The acid was neutralized by washing the cells in 0.1 M sodium borate buffer (pH 8.5) for 10 min and then washing the cells again in PBS. Fixed cells were blocked in 5% normal goat serum with 0.3% Triton X-100 and incubated with anti-BrdU antibodies (rat monoclonal, 1∶500, Accurate Chemical, Westbury, NY) for 30 min. To label neurons or astrocytes, fixed cells were incubated for 30 min with primary antibodies to ß-tubulin-III (Tuj1; mouse monoclonal IgG2b, 1∶500, Sigma-Aldrich) and glial fibrillary acidic protein (GFAP; rabbit polyclonal, 1∶1,000, DAKO, Glostrup, Denmark), respectively.

After incubation with the primary antibodies, the cultures were rinsed in PBS, and incubated for 30 min with secondary antibody conjugated to Cy3 or Alexa Fluor 488 (goat anti-IgG, 1∶1000, Jackson ImmunotechResearch Laboratories, West Grove, PA). Hoechst 33258 (0.5 µg/ml in PBS, Sigma-Aldrich) was added for 10 min after completion of the secondary antibody incubation as a nuclear stain. Cell counts were performed using a Nikon fluorescence microscope (Nikon, Tokyo, Japan) and Metamorph Imaging software (Universal Imaging Corporation, Downington, PA). Quantification of cells was based on counting the number of Hoechst-stained nuclei and the specific immunostained cells in at least four independent fields (total area>25 mm^2^) from a minimum of three coverslips.

### Western Blotting and Phosphorylation Assay

Western blot was used to measure the protein expression of two known cell proliferation markers, proliferation cell nuclear antigen (PCNA) and cyclin-dependent kinase (CDK1/cdc2), and to estimate the activation of intracellular signaling cascade by detecting the phosphorylation of extracellular signal-regulated kinase (pERK). Neurospheres were lysed using ice-cold lysis buffer as described previously [19]. Total protein concentrations were determined by using a range of bovine serum albumin standards in conjunction with a BioRad protein analysis kit (BioRad, Hercules, CA). Cell protein lysate (10–20 µg) was separated under reducing and denaturing conditions on 12% sodium dodecyl sulfate-polyacrylamide gel electrophoresis (SDS-PAGE) and electrotransferred to a polyvinylidone difluoride membrane (GE Water & Process Technologies, Trevose, PA). Nonspecific binding sites were blocked with 5% skim milk in Tris-buffered saline containing 0.05% Tween 20. Membranes were blotted using an antibody for PCNA (mouse monoclonal; 1∶300, clone PC10; Zymed Laboratories Inc., San Francisco, CA), carboxy-terminal domain of CDK1/cdc2 (rabbit polyclonal; 1∶500; Novus Biologicals Inc., Littleton, CO), human specific SOD1 (rabbit polyclonal, 1∶500; EMD Millipore, Billerica, MA) and ß-actin (rabbit polyclonal; 1∶200; Sigma-Aldrich). For measuring the pERK expression, an anti-pERK1/2 antibody (rabbit polyclonal; 1∶500; Sigma-Aldrich) was used. The membranes were then incubated in horseradish peroxidase-conjugated anti-rabbit (1∶5000; Promega Corp., Madison, WI) or mouse IgG (1∶1000; IMGENEX. San Diego, CA) and the results were visualized using an Amersham enhanced chemiluminescent (ECL) and western blotting detection kit (GE Healthcare, Piscotoway, NJ). The blots were semi-quantitated by optical density analysis using Image J software. Three independent rNPC lines in each group were used for densitometric analysis. The data were expressed as means ± SEM.

### Cell Viability Assay

Neurospheres were dissociated and plated on a 24-well plate as described above. After 24 hours, the cells were exposed to 1 mM H_2_O_2_ (Sigma-Aldrich) for 15 minutes or 5 mM 3-morpholinosydnonimine (SIN-1; R&D Systems) for 2 hours and then washed and maintained in the differentiation medium for an additional 24 hours. Then, the conditioned media was collected and its cytotoxicity was measured via lactate dehydrogenase (LDH) release as directed by the manufacturer's protocol (Cyto Tox 96 Non-Radioactive Cytotoxic Assay kit, Promega). The optimal density of each sample was measured using a Molecular Devices SpectraMax 340 pc system at 490 nm. To determine the percentage of cytotoxicity, experimental LDH release was divided by maximum LDH release.

### Statistical Analysis

All data were expressed as means ± SEM and were analyzed using one-way functional ANOVA using Newman-Keuls *post hoc* comparisons (Graphpad Prizm software, San Diego, CA). All differences were considered significant when P<0.05.

## Results

### Preparation of male and female rNPCs over-expressing mutant SOD1 gene

In the present study, we had two major questions: 1) do male and female rNPCs have different levels of cell proliferation, neurogenesis, and cell viability after being exposed to oxidative stress and 2) do rNPCs over-expressing mutant SOD1 gene show different results? To answer these questions, primary E14 rat fetuses were harvested from pregnant SOD1^G93A^ transgenic rats. The spinal cord and cortex from individual animals was dissected and genotyped by PCR with primers for the mutant SOD1^G93A^ gene or the male specific *Sry* gene (Fig. 1A). First, we confirmed these primers could specifically amplify these two genes using tail snip samples from adult SOD1^G93A^ rats (Fig. 1B). We then genotyped the embryonic tissues and formed four groups: 1) male SOD1^G93A^ (−), 2) male SOD1^G93A^ (+), 3) female SOD1^G93A^ (−), and 4) female SOD1^G93A^ (+) (Fig. 1A, C). The tissues within each group were pooled and chopped into small cubes before being transferred to maintenance media containing EGF and FGF-2. Round aggregates of cells termed neurospheres began to form as described previously [18], [25].

### Effects of mutant SOD1 gene and sex in cell proliferation of rNPC

**Figure 2:**
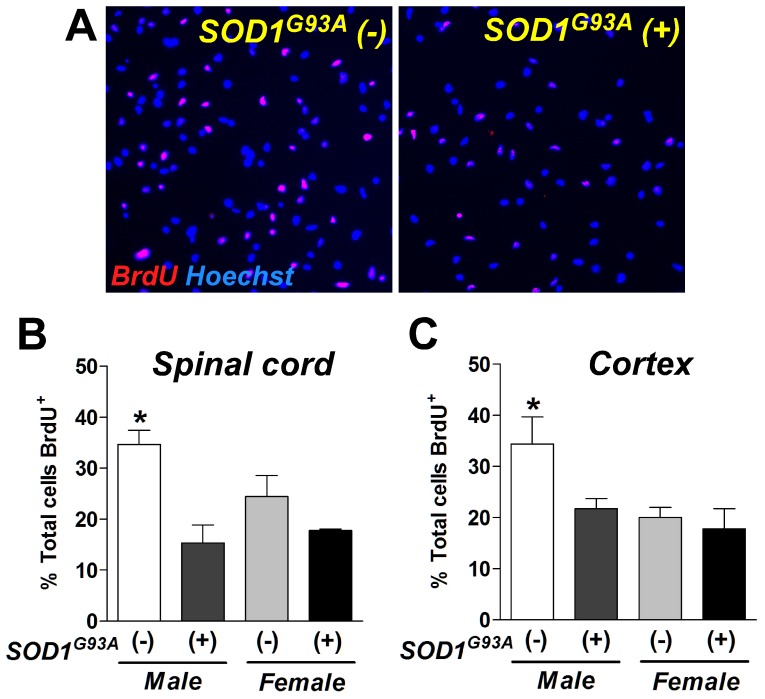
Over-expression of the mutant SOD1 gene significantly reduces proliferation of rat NPCs. (**A**) Photomicrographs represent BrdU immunoreactivity in wild type [SOD1 (−)] or transgenic [SOD1 (+)] NPC in male cells derived from the spinal cord. (**B and C**) The number of BrdU positive cells was decreased in male SOD1^G93A^ (+) cells derived from the spinal cord (**B**) and cortex tissues (**C**). *: P<0.05 vs. other groups.

After preparing the four rNPC lines described previously, we first asked whether over-expression of the mutant SOD1 gene affects proliferation in neurospheres. Cell proliferation was assessed in neuropheres using 5-bromo-2′-deoxyuridine (BrdU) incorporation [19], [24]. Neurospheres were pulse-labeled with BrdU for 14 hours, dissociated, plated for 1 hour, and immunostained with anti-BrdU antibodies (Fig. 2A). In the neurospheres derived from the spinal cords (Fig. 2A and B), the wild-type male rNPCs [male SOD1^G93A^ (−); n = 4] showed more BrdU-postive cells when compared to the female SOD1^G93A^ (−) cells (P<0.05; n = 4). This indicates that there was a sexual difference in proliferation rate. Male SOD1^G93A^ (+) rNPCs (n = 4) showed a significant reduction of BrdU incorporation from 37% to 25% when compared to male SOD1^G93A^ (−) cells (n = 4) (Fig. 2A, B). However, the SOD1^G93A^ transgene did not affect cell proliferation in female cells. The cortical tissue-derived neurospheres showed similar trends (Fig. 2C). These data suggest that the SOD1^G93A^ transgene and sex affect the division of the cells within neurospheres.

### Neurogenic effects of mutant SOD1 gene and sex on rNPCs

**Figure 3:**
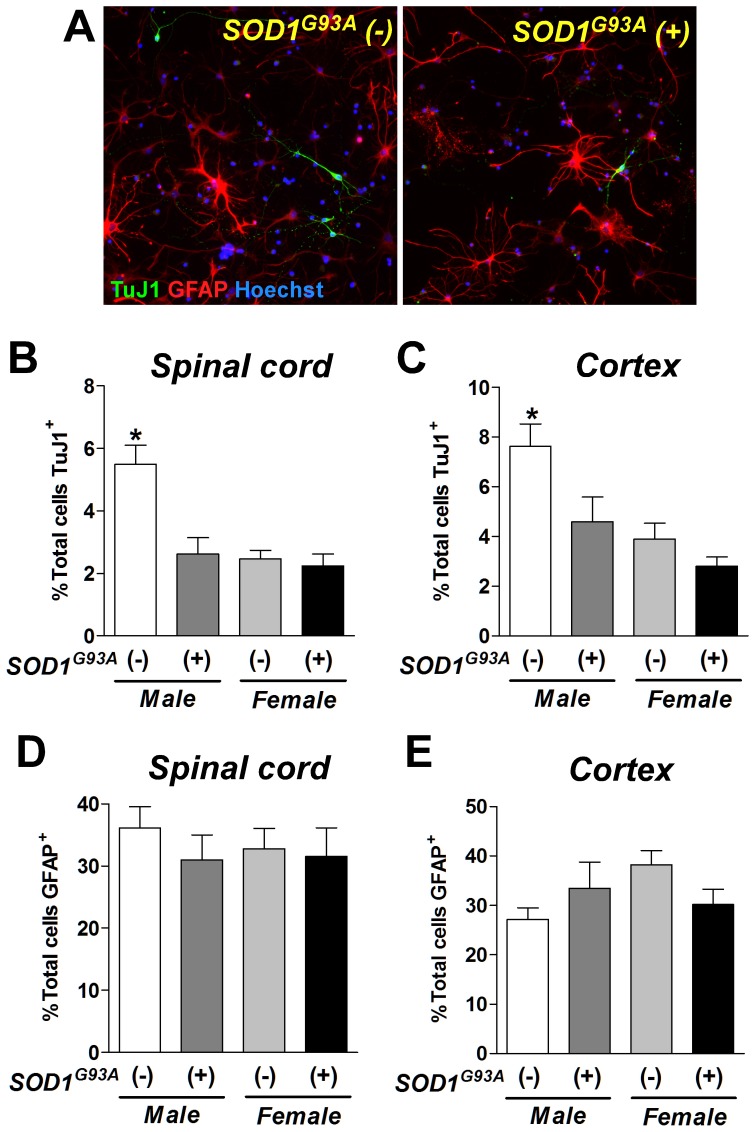
The number of mature neurons was decreased in differentiated cells with SOD1^G93A^ transgene. (**A**) After 7 days of differentiation, TuJ1 (green) and GFAP (red)-positive cells were found in male wild type [SOD(−)] or transgenic [SOD(+)] cells. The number of TuJ1-postive neurons was significantly decreased by SOD1^G93A^ over-expression in male cells derived from the spinal cord (**B**) and cortex (**C**). There was no difference in the number of GFAP-positive astrocytes differentiated from the spinal cord (**D**) and cortex (**E**) rNPCs. *: P<0.05 vs. other groups.

We next hypothesized that over-expression of the mutant SOD1 gene and sex would affect the level of neural differentiation *in vitro*. Four groups of neurospheres (n = 4 each group) were dissociated, plated onto coverslips, and allowed to differentiate for 7 days in serum-free conditions. The differentiated cells were immunostained for β-tubulin III (TuJ1; a specific marker for neurons) and glial fibrillary acidic protein (GFAP; a marker for astrocytes). The number of TuJ1-positive neurons was significantly different between the wild-type male and female cells (P<0.05). The number of TuJ1 positive cells was significantly reduced in the male SOD1^G93A^ (+) lines from 5.5% to 2.4% compared to the male SOD1^G93A^ (−) cells (Fig. 3A, B). However, over-expression of mutant SOD1 gene did not affect neurogenesis in female cells. Similar results were observed with the cortex-derived neurospheres (Fig. 3C). In contrast to TuJ1 positive cells, the number of GFAP positive cells was not significantly different between the groups (Fig. 3D, E). These results indicate that male cultures produce more neurons compared to females and SOD1^G93A^ over-expression significantly reduces the number of neurons after differentiation.

### Effects of sex and the SOD1^G93A^ transgene on the expression of cell cycle proteins and the activation in rNPCs

**Figure 4:**
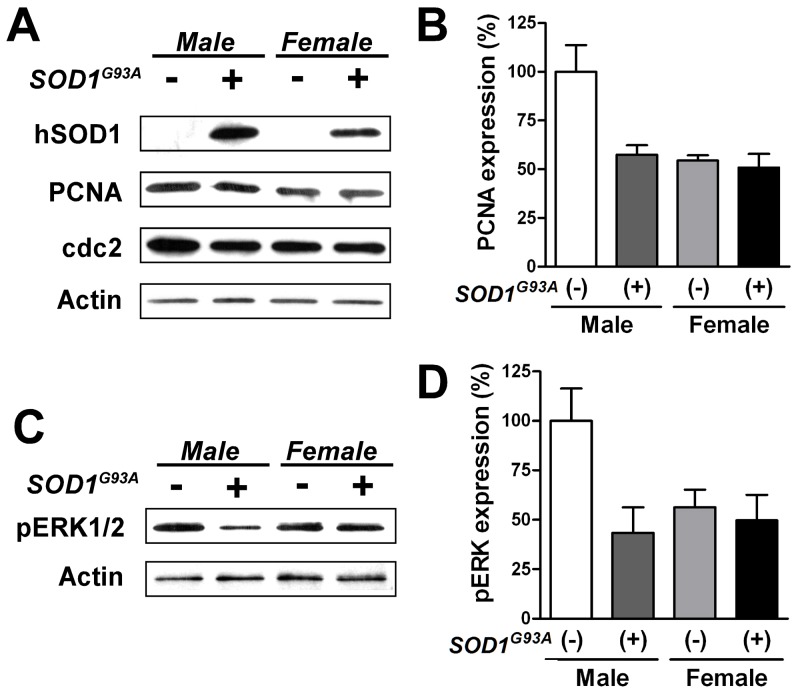
Expression of cell cycle marker proteins was reduced in male SOD1^G93A^ rNPCs. (**A**) Western blot analysis was used to measure the protein expression of proliferating cell nuclear antigen (PCNA) and cyclin-dependent kinase (cdc2), two known cell proliferation markers. (**B**) Semi-quantitative densitometric analysis of PCNA protein expression. (**C and D**) Semi-quantitative densitometric analysis using Western blotting revealed that SOD1^G93A^ transgene altered the phosphorylation of extracellular signal-regulated kinase 1/2 (pERK1/2) in male rNPCs. Three independent rNPC lines in each group were used for densitometric analysis. The data were expressed as means ± SEM.

In order to confirm the results from the BrdU incorporation assay, we determined the absolute protein levels of proliferating cell nuclear antigen (PCNA) and cyclin-dependent kinase (CDK1/cdc2), two known cell proliferation markers, using Western blot (Fig. 4A). In the neruospheres derived from fetal spinal cord, male SOD1^G93A^ (−) expressed twice as much PCNA as the other rNPC lines (Fig. 4B). Similar densitometric results were observed with cdc2 expression (data not shown). Additionally, we confirmed expression of the human mutant SOD1 protein in both male and female SOD1^G93A^ (+) neurospheres (Fig. 4A). We also estimated the activation of intracellular signaling cascades by detecting phosphorylation of ERK1/2 (pERK1/2) (Fig. 4C). Densitometric analysis revealed that male SOD1^G93A^ (−) showed twice the level of pERK1/2 compared to the other groups (Fig. 4D).

### Effects of sex and the SOD1^G93A^ transgene on responses to exogenous oxidative stress

**Figure 5:**
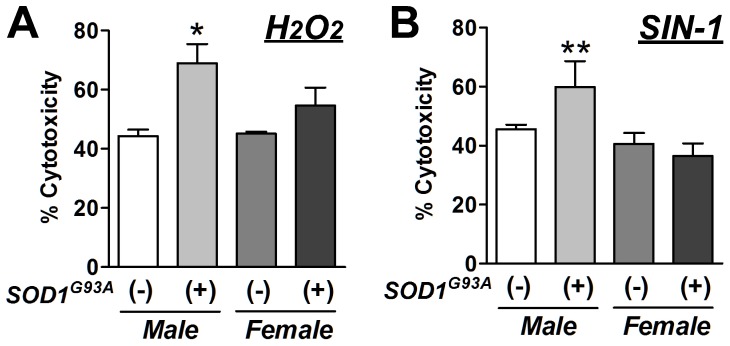
SOD1^G93A^ over-expressing rNPCs were more sensitive to oxidative stress. H_2_O_2_ (**A**) and SIN-1 (**B**) significantly increased the LDH released into the media from the cells derived from SOD1 transgenic males compared to the wild type male cells. *: P<0.01 and **: P<0.05 vs. wild-type male.

We next hypothesized whether over-expression of the mutant SOD1 gene and sex affected the rNPCs' sensitivity to oxidative stress. Four different groups of neurospheres (n = 4 each group) were dissociated and plated down on 24-well plates. After 24 hours, the cells were exposed to 1 mM hydrogen peroxide (H_2_O_2_) for 15 minutes or 5 mM 3-morpholinosydnonimine (SIN-1), a free radical-generating agent, for 2 hours. Then, the cells were washed and maintained in the differentiation medium for an additional 24 hours. The conditioned media was collected, and cytotoxicity was measured via lactate dehydrogenase (LDH) release. LDH is released from dying cells or cells with a compromised cell membrane and is commonly used as a marker of cell death. H_2_O_2_ (Fig. 5A, P<0.05) and SIN-1 (Fig. 5B, P<0.01) significantly increased the amount of LDH released into the media from male SOD1^G93A^ (+) neurosphere cells compared to male SOD1^G93A^ (−) cells. However, this phenomenon was not observed in female cells. Taken together, these results support our hypothesis that over-expression of the mutant SOD1 gene and sex affect cells' vulnerability to oxidative stress in culture.

## Discussion

In this study, we prepared neurospheres with the mutant SOD1 gene to model familial ALS and showed that sex and SOD1 over-expression play a critical role in proliferation, neurogenesis and sensitivity to oxidative stress in rNPCs. Neurosphere culture is recognized as an excellent technique with regard to isolating neural progenitor/stem cells to investigate the differentiation potential of cell lineages because of its flexibility in dissociation and expansion. At a cellular level, this is the first report to show 1) sexual dimorphism pertaining to cell proliferation and neurogenesis in rNPCs, and 2) that the mutant SOD1 gene reduces cell division and neurogenesis and increases sensitivity to exogenous oxidative stress in male, but not female, rNPCs.

The present data support the idea that mitotic and neurogenic differences occur in a sex-dependent manner in ALS. Sexual dimorphism has already been described regarding various functional aspects in different types of stem cells [26]–[30]. In particular, sexual dimorphism in the neurogenic capacity was observed in cultured NPCs derived from the subventricular zone of adult rats [29], [30]. The same studies also showed that the expression of estrogen receptors and aromatase, a key enzyme to metabolize testosterone to 17β-estradiol, were different between male and female NPCs [29], [30]. Different expression levels of steroid receptors and metabolizing enzymes may also contribute to the sexual dimorphism observed regarding cell proliferation and neurogenesis in fetal-tissue derived rNPCs. Sexual dimorphism in stem cells could be the result of sex steroids, but it might also be caused by the intrinsic differences between sex chromosomes in male and female NPCs. In mammals, the Y chromosome encodes male-specific genes such as *Sry* [31]. During testis development, SRY protein has been known to up-regulate the expression of Sox9 [32], which is also expressed in neural stem cells in the CNS and recognized as a key transcription factor involved in altering neurogenic potential in these cells [33]. Further profiling the expression of steroid receptors and Sox proteins would help determine the molecular mechanism of sexual dimorphism in neural progenitor/stem cells.

Over-expression of the mutant SOD1 gene affected cell proliferation in undifferentiated rNPCs. A similar effect was observed in a recent study using NSC-34 neuronal cells over-expressing the SOD1^G093A^ gene [34]. An *in vivo* study using adult SOD1^G93A^ mice showed that the number of BrdU-pulse labeled cells was significantly decreased in the forebrain regions [35]. On the other hand, Chi *et al.* [36] reported that there was an increase in NPC proliferation, migration, and neurogenesis in the adult spinal cord of bi-transgenic mice containing both nestin promoter-driven LacZ reporter and SOD1^G93A^ genes. Our *in vitro* results provided more direct evidence of the effects of the mutant SOD1 gene, although further studies are necessary to explore the effects of mutant SOD1 over-expression on cell proliferation in NPCs at development and postnatal stages. Additionally, reduced proliferation due to SOD1^G93A^ over-expression was more pronounced in male cells compared to female cells. Therefore, proliferating male cells would be more susceptible to the toxic effects of mutant SOD1^G93A^ proteins.

SOD1^G93A^ over-expression significantly decreased neurogenesis, but not astrocyte differentiation, more so in male cells after differentiation. Since our rNPCs were derived from the fetal CNS, the present results indicate that the SOD1^G93A^ transgene may play a key role in modifying the number of neurons and neural differentiation in the male spinal cord during development. When cultured NPCs can be differentiated into various neurons and glial cells, the majority of the differentiated neurons are similar to interneurons with small nuclei and bipolar morphology [19], [23], [37]. Interestingly, recent studies have suggested that the loss of or abnormalities in inhibitory interneurons contribute to ALS pathogenesis [38]. The intermediate zone of the spinal cord contains several neuron types that play important roles in controlling the activities of motor neurons. There are reports of losses of intermediate zone interneurons in the spinal cord of ALS patients [39] and SOD1^G93A^ transgenic mice [40]. Taken together with our current results, the mutant SOD1 gene and/or sex may alter the number and characteristics of interneurons during development in the central nervous system. These anatomical changes may cause sexual dimorphism during the disease course of ALS. To prove this hypothesis, the role of interneurons during development and their specific abnormalities in ALS need to be explored.

There is a great deal of data supporting the involvement of oxidative stress in ALS pathogenesis. Analysis of post mortem tissue from ALS patients has revealed increased oxidative damage to cellular components, such as oxidized DNA and the formation of carbonyl and nitrotyrosine derivatives in proteins, compared to control groups. This is consistent with previous results using primary culture cells derived from ALS patients. Greater sensitivity to oxidative stress was observed in fibroblasts from familial ALS patients with a SOD1 mutation [41]. Another study demonstrated that exposure to paraquat, an oxidative stress-inducing reagent, increased apoptosis in cultured myoblasts derived from familial ALS patients [42]. In the current study, we used rNPCs and showed that mutant SOD1 over-expression increased sensitivity to oxidative stress. Furthermore, this effect was more pronounced in male rNPCs compared to female cells. Together, all these data support the hypothesis that oxidative stress contributes to abnormalities in neuronal and non-neuronal cells during ALS pathogenesis.

Although we provided direct and quantitative evidence of sexual differences in rNPCs with mutations in the SOD1 gene, the underlying mechanisms of sexual differences in ALS pathology are uncertain at this time. It has been broadly recognized that sex appears to modify the course of disease progression in animals with mutations in the SOD1 gene and ALS patients [6]. However, the causes of these sexual differences are still a mystery in ALS pathogenesis. Previous reports using SOD1^G93A^ mice suggested that sex steroids are involved in the occurrence and disease progression of ALS [10], [14]. The potential role of gonadal steroids was supported by recent studies using SOD1^G93A^ mice. Ovariectomy accelerated disease progression in females and subsequent administration with estrogen reversed the harmful effects of the ovariectomy [10], [14]. On the other hand, our latest study using SOD1^G93A^ rats indicated that gonadal steroids are not a key modulator of the occurrence or disease progression in a rat model of ALS [11]. Our *in vitro* studies support the hypothesis that sexual dimorphism in ALS is caused by intrinsic cellular differences of sex and the SOD1^G93A^ transgene. Further analysis is necessary to understand how sexual dimorphism is involved in ALS disease progression.

In conclusion, our study demonstrates that over-expression of the mutant SOD1 gene and sex affected the level of proliferation, neural production after differentiation, and sensitivities to oxidative stress in rNPCs. Rat neurosphere culture provided new information on how the mutant SOD1 gene and sexual dimorphism are involved in ALS disease progression.
